# Influence of minimal invasive extracorporeal circuits on dialysis dependent patients undergoing cardiac surgery

**DOI:** 10.1177/02676591231216794

**Published:** 2023-11-17

**Authors:** Thai Duy Nguyen, Mohammed Morjan, Khaldoun Ali, Ingo Breitenbach, Wolfgang Harringer, Aschraf El-Essawi

**Affiliations:** 1 Clinic for Pediatric & Congenital Heart Surgery, Children’s Heart Center, 39058 University Hospital RWTH Aachen, Germany; 2 Department of Cardiovascular Surgery, 39064 University Hospital Düsseldorf, Germany; 3 Department of Thoracic and Cardiovascular surgery, 39726 Braunschweig Municipal Hospital Germany; 4 Department of Thoracic and Cardiovascular surgery, 27177 University Medical Center Göttingen, Germany

**Keywords:** MiECC, cardiopulmonary bypass, cardiac surgery, dialysis, end stage renal disease, extracorporeal circulation, minimal invasive

## Abstract

**Introduction:**

Cardiac surgery in patients on chronic renal dialysis is associated with significant morbidity and mortality. Minimally invasive extracorporeal circuits (MiECC) have shown a positive impact on patient outcome in different high-risk populations. This retrospective study compares the outcome of these high-risk patients undergoing heart surgery either with a MiECC or a conventional extracorporeal circulation (CECC).

**Methods:**

This is a single-center experience including 131 consecutive dialysis dependent patients undergoing cardiac surgery between January 2006 and December 2016. A propensity score matching was employed leaving 30 matched cases in each group.

**Results:**

After propensity score matching the 30-day mortality was significantly lower in the MiECC group (*n* = 3 (10%) vs *n* = 10 (33%) in the CECC group, *p* = .028). Further, intraoperative transfused units of packed red blood cells were lower in the MiECC group (1.4 ± 1.8 units vs 2.8 ± 1.7, *p* < .001).

**Conclusions:**

There are evident advantages to using MiECC in dialysis dependent patients, especially regarding mortality. These findings necessitate additional research in MiECC usage in high-risk populations.

## Introduction

The benefits of minimal invasive extracorporeal circuits (MiECC) over conventional heart-lung machines have been demonstrated in terms of perioperative blood conservation,^1–4^ incidence of postoperative atrial fibrillation,^3,5,6^ and improved renal and myocardial protection,^7^ resulting in an overall reduction in postoperative morbidity and mortality.^2,8–10^ This favourable effect was especially evident in octogenarians, who underwent coronary artery bypass grafting with or without aortic valve replacement.^2,10^ We believe that this positive impact relates to a better preservation of homeostasis and hence of physiologic reserves that are of paramount importance for high-risk patients to overcome the perioperative burdens of cardiac surgery.

Patients on chronic renal dialysis who are in need of cardiac surgery constitute a similarly high-risk cohort with depleted physiologic reserves in whom surgery is associated with significant morbidity and mortality. We hence hypothesized that in this subpopulation of high-risk patients, MiECC may demonstrate similar positive effects on patient outcome. Although there are indications that MiECC may be superior to CECC in dialysis-dependent patients undergoing cardiac surgery, a comparative study has not yet been published. This study aimed to provide such a comparison.

## Patients and methods

This retrospective, single-center analysis included all dialysis dependent patients who underwent cardiac surgery in our department between January 2006 and December 2016. Reoperations and minimally invasive cases (both were by far more commonly performed by CECC), as well as off-pump bypass surgeries were excluded. The local ethics committee (Medizinische Hochschule Hannover) approved the study (reference no. 9200_BO_K_2020).

## Procedural strategy

### MiECC

A modular closed-loop circuit system with an arterial filter, bubble detector and bubble trap was employed (Type IV).^11^ Sarns™ Centrifugal Pumps and CAPIOX® RX15 Oxygenators by Terumo Corporation (Tokyo, Japan) were utilized. Quick connectors with a hard-shell reservoir could be introduced to the venous line in case a conversion to an open circuit was needed. Priming was achieved with 600 mL crystalloid solution. A cell-saver was utilized in each procedure.

### CECC

We used a standard open circuit with a hard-shell reservoir and cardiotomy suction. 1500 mL of crystalloid solution was used to prime the circuits. After heparin reversal a cell-saver was utilized in case of major bleeding.

Autologous priming was used in the majority of patients. Myocardial protection was established with intermittent warm blood cardioplegia in MiECC patients unless double venous cannulation was warranted in which case Bretschneider cardioplegia was utilized. On the other hand, Bretschneider cardioplegia was utilized in all CECC patients. In all cases in whom double venous cannulation was utilized the Bretschneider Cardioplegia was drained via the right atrium. Heparinisation was achieved with a target activated clotting time of 480 s. After weaning from bypass, protamine was administered with a ratio of heparin: protamine 1: 0.8. All patients had the same anaesthesiologists, surgeons, and perfusionists, as well as the same anaesthetic management.

Although MiECC systems have evolved over the last decade, our MiECC setup over the whole study period did not change as the ROCsafe® system was developed as a modular system from the start. Additional information on the extracorporeal circuits utilized were previously published.^12^

A liberal transfusion strategy was adopted in this patient collective, with a hemoglobin level below approximately 10 g/dl as a trigger for red blood cell transfusions. Fresh frozen plasma and platelets were substituted based on amount of bleeding and traditional lab results, which has evolved to ROTEM based assessments in later years. However, transfusions were always guided by clinical pathways which were always independent of the type of extracorporeal circuit utilized.

## Data collection and definitions

Patient information were retrieved from our departments database. These included demographics, baseline characteristics, EuroSCORE I, type of dialysis, duration of dialysis, operative data and postoperative course. The definitions of the variables that are included in the EuroSCORE I were adopted. Stroke was defined as any focal or global neurological deficit persisting for more than 24 h and confirmed by CT scan or MRI. Myocardial infarction was defined as CKMB five times or more above the normal upper reference limit within the first 72 h after surgery or above the upper reference limit thereafter in combination with clinical symptoms of ischemia or ECG changes or angiographic evidence of new graft or native coronary artery occlusion. Pneumonia was defined as postoperative rise of inflammatory markers, clinical symptoms and correlating chest X-ray findings. Sepsis was defined as any infection with an increase of 2 SOFA points. Resuscitation was defined as postoperative need for basic life support secondary to cardiac arrest or ventricular fibrillation. Low cardiac output was diagnosed if a patient required mechanical circulatory support to be weaned from cardiopulmonary bypass or because of hemodynamic compromise on ICU. It was also diagnosed if a patient required inotropic support to maintain a cardiac index above 2.0 L/min/m^2^ despite optimal fluid balance. Postoperative delirium was defined as a sudden alteration of the consciousness including agitation, hallucination, memory loss and/or loss of attentiveness which required drug treatment. New onset atrial fibrillation was defined as any arrhythmia persisting for >30 s and showing the following electrocardiographic features: irregular R– R intervals when atrioventricular conduction is present, absence of distinct and repeat P waves and irregular atrial activity, if no prior arterial fibrillation has been diagnosed before. Major adverse events were defined as myocardial infarction, low cardiac output, severe infection including pneumonia, mediastinitis or sepsis, stroke, rethoracotomy, resuscitation, or death.

## Statistical analysis

Student’s t-test was applied to continuously distributed variables presented as mean standard deviation and Mann-Whitney U Test was employed when appropriate. To compare categorial variables, Fisher’s exact test or the chi-square test were utilized as required. A *p* value of 0.05 or less was considered significant. The study was conducted using version 29.0 of the statistical application IBM SPSS (IBM Corp. Armonk, NY).

## Propensity score matching

A propensity score analysis with a multivariable logistic regression model was performed on the whole collective to limit the influence of confounding factors. The type of circuit was the dependent variable, whereas all relevant preoperative and intraoperative factors (sex, hypercholestremia, peripheral vascular disease, diabetes mellitus, systemic hypertension, EuroSCORE I, concomitant procedure, isolated valve, isolated CABG, bypass time and clamping time) were utilized as covariates to generate the propensity score for each patient. These scores were used to match patients who had a MiECC with patients who received a CECC. As the maximum caliper width for matching the two treatment groups a 0.2 difference in propensity score was used.

## Results

Based on the time span and selection, a total of 131 patients were included in this study. Table 1 provides a summary of the demographics and clinical characteristics of the whole population.

**Table 1.** Baseline characteristics of whole patient collective.

Variable	All patients = 131	MiECC = 63	CECC = 68	*p*-value
Age (years)	66 ± 10.9	66 ± 10.7	66 ± 11.1	0.969
Males *n* (%)	99 (76)	54 (86)	45 (67)	0.013
BMI (kg/m^2^)	27 ± 5.1	26 ± 4.3	27 ± 5.7	0.705
EF (%)	49 ± 13	49 ± 12.7	48 ± 13.3	0.591
COPD *n* (%)	15 (12)	7 (11)	8 (12)	0.907
Hyperlipidemia *n* (%)	58 (44)	34 (54)	24 (35)	0.032
Peripheral vascular disease *n* (%)	51 (39)	39 (48)	21 (31)	0.050
Diabetes mellitus *n* (%)	59 (45)	33 (52)	26 (38)	0.104
Systemic hypertension *n* (%)	108 (84)	56 (89)	52 (80)	0.166
Pulmonary hypertension *n* (%)	15 (12)	6 (10)	9 (13)	0.505
Urgent surgery *n* (%)	48 (37)	21 (33)	27 (40)	0.449
Emergent surgery *n* (%)	22 (17)	12 (19)	10 (15)	0.507
Log. EuroSCORE I	14.7 ± 12.9	12.1 ± 10.5	17.2 ± 14.5	0.020
Time on dialysis (months)	50 ± 49	48 ± 47	52 ± 51	0.862
PD *n* (%)	23 (18)	12 (19)	11 (16)	0.666
CABG *n* (%)	64 (49)	48 (76)	16 (24)	<0.001
Valvular surgery *n* (%)	32 (24)	6 (10)	26 (38)	<0.001
CABG + valve *n* (%)	35 (27)	9 (14)	26 (38)	0.002

Data are presented as mean ± SD or number (%).

EF = Ejection fraction, BMI = Body-Mass-Index, COPD = Chronic obstructive pulmonary disease, EuroSCORE = European System for Cardiac Operative Risk Evaluation, PD = Peritoneal dialysis, CABG = Coronary artery bypass grafting, CABG + Valve = combined procedure. Definition of the variables that appear in the Euroscore were adopted accordingly.

After employing the propensity score matching, 60 patients were matched and analysed, Table 2 depicts their preoperative characteristics. A logistic EuroSCORE I of 14.6 ± 12.2% and a high prevalence of urgent and emergent operations *n* = 33 (54%) is noteworthy. Furthermore, concerning the MiECC cohort, not a single case had to be converted to an open system.

**Table 2.** Baseline characteristics of propensity score matched patients.

Variable	All patients = 60	MiECC = 30	CECC = 30	*p*-value
Age (years)	67 ± 11.4	64 ± 11.6	68 ± 11	0.133
Males *n* (%)	45 (75)	23 (77)	22 (73)	0.766
BMI (kg/m^2^)	28 ± 5.3	27 ± 4.5	28 ± 6	0.755
EF (%)	49 ± 13.5	48 ± 12.7	49 ± 14.5	0.747
COPD *n* (%)	8 (13)	4 (13)	4 (13)	1.000
Hyperlipidemia *n* (%)	23 (38)	13 (43)	10 (33)	0.426
Peripheral vascular disease *n* (%)	18 (30)	10 (33)	8 (27)	0.573
Diabetes mellitus *n* (%)	25 (42)	14 (47)	11 (37)	0.432
Systemic hypertension *n* (%)	46 (77)	25 (83)	21 (70)	0.222
Pulmonary hypertension *n* (%)	7 (12)	4 (13)	3 (10)	1.000
Urgent surgery *n* (%)	22 (37)	10 (33)	12 (40)	0.592
Emergent surgery *n* (%)	11 (18)	8 (27)	3 (10)	0.095
Log. EuroSCORE I	14.6 ± 12.2	12.3 ± 10.4	16.9 ± 13.5	0.132
Time on dialysis (months)	59 ± 52	68 ± 54	50 ± 49	0.126
PD *n* (%)	10 (17)	7 (23)	3 (10)	0.166
CABG *n* (%)	31 (52)	16 (53)	15 (50)	0.796
Valvular surgery *n* (%)	10 (17)	6 (20)	4 (13)	0.488
CABG + valve *n* (%)	19 (32)	8 (27)	11 (37)	0.405

Data are presented as mean ± SD or number (%).

EF = Ejection fraction, BMI = Body-Mass-Index, COPD = Chronic obstructive pulmonary disease, EuroSCORE = European System for Cardiac Operative Risk Evaluation, PD = Peritoneal dialysis, CABG = Coronary artery bypass grafting, CABG + Valve = combined procedure. Definition of the variables that appear in the Euroscore were adopted accordingly.

Intraoperative results showed a significant difference between the two arms regarding transfused units of packed red blood cells (1.4 ± 1.8 vs. 2.8 ± 1.7 units, *p* < .001) and the number of transfused patients (15 (50%) vs. 28 (93%) patients, *p* < .001) (Table 3). While a significant difference was observed in 30-day mortalities (3 vs. 10 patients, *p* = .028) (Table 4), differences in the overall transfusions for red blood cells, fresh frozen plasma or platelets throughout the hospital stay did not.

**Table 3.** Intraoperative outcomes propensity score matched patients.

Variable	All patients = 60	MiECC = 30	CECC = 30	*p*-value
Clamping time	76 ± 42	68 ± 36	84 ± 46	0.067
Bypass time	126 ± 69	114 ± 66	137 ± 72	0.071
Transfused RBC units	2.1 ± 1.9	1.4 ± 1.8	2.8 ± 1.7	<0.001
Transfused patients (RBC) *n* (%)	43 (72)	15 (50)	28 (93)	<0.001
Transfused FFP units	1.2 ± 2.2	0.8 ± 1.6	1.6 ± 2.6	0.213
Transfused patients (FFP) *n* (%)	18 (30)	7 (23)	11 (37)	0.260
Transfused platelet units	0.2 ± 0.6	0.2 ± 0.6	0.3 ± 0.7	0.920
Transfused patients (platelets) *n* (%)	8 (13)	4 (13)	4 (13)	1.0

Data are presented as mean ± SD. Times in minutes. RBC = Red blood cell, FFP = fresh frozen plasma concentrate.

**Table 4.** In-hospital outcomes propensity score matched patients.

Variable	All patients = 60	MiECC = 30	CECC = 30	*p*-value
30-day mortality	13 (22)	3 (10)	10 (33)	0.028
Adverse events^a^	22 (37)	9 (30)	13 (43)	0.284
Myocardial infarction	1 (2)	0	1 (3)	1.0
Reoperation	2 (3)	0	2 (7)	0.492
Pneumonia	3 (5)	1 (3)	2 (7)	1.0
Stroke	0	0	0	0
Mediastinitis	1 (2)	0	1 (3)	1.0
Resuscitation	3 (5)	2 (7)	1 (3)	1.0
Sepsis	3 (5)	1 (3)	2 (7)	1.0
Low cardiac output	5 (8)	0	5 (17)	0.052
Postoperative delirium	5 (8)	4 (13)	1 (3)	0.353
NOAF	13 (22)	5 (17)	8 (27)	0.347
Transfused RBC units	3.8 ± 3.5	3.4 ± 2.9	4.3 ± 3.9	0.226
Transfused patients (RBC) *n* (%)	54 (90)	26 (87)	28 (93)	0.424
Transfused FFP units	3 ± 5	2.7 ± 3.7	3.4 ± 6.1	0.918
Transfused patients (FFP) *n* (%)	31 (52)	15 (50)	16 (53)	0.796
Transfused platelet units	0.3 ± 1	0.2 ± 0.7	0.4 ± 1.2	0.930
Transfused patients (platelets) *n* (%)	8 (13)	4 (13)	4 (13)	1.0

Data are presented as mean ± SD or number (%).

NOAF = New onset atrial fibrillation, RBC = Red blood cell, FFP = Fresh frozen plasma concentrate.

^a^Adverse events were defined as infarction, low cardiac output, stroke, pneumonia, reoperation, resuscitation, death, mediastinitis, sepsis or postoperative delirium.

A total of 22 patients experienced major adverse events in the matched population, 9 in the MiECC group and 13 in the CECC group. The most common adverse events were 30-day mortality with 22.7% as well as postoperative delirium and low cardiac output which each occurred in 10.7% of cases. It is noteworthy that none of the MiECC patients experienced a low cardiac output postoperatively (Table 4).

The most prevalent cause of death was low cardiac output (*n* = 4), which accounted for 31% of deaths. Other causes of death were pneumonia (*n* = 2), multi-organ failure (*n* = 2), ventricular tachycardia (*n* = 1), sepsis (*n* = 1), electromechanical decoupling (*n* = 1) and myocardial infarction (*n* = 1). In one patient the cause of death could not be determined, it occurred after hospital discharge. Of the 3 patients who died in the MiECC group 1 had an isolated CABG and 2 had combined procedures. 5 of the 10 patients who died in the CECC group had an isolated CABG, 3 had an isolated AVR and 2 had a combined procedure.

The intra- and postoperative outcomes of the whole population are summarized in Table S1 and S2 in the supplement.

## Discussion

In 2017, the number of patients requiring dialysis in Germany was estimated in a projected study to be approximately 100.000, with a prognosed increase of 20–23% by 2040.^13^ We therefore expect a similar increase in the number of patients presenting for cardiac surgery within this population within the next two decades. As evidence shows that the mortalities among this patient population are 2–4-fold higher than in patients with normal renal function there is a dire need to improve outcomes in these high-risk patients.^14–16^

For this population, early mortality rates of 9.4 to 20.8% have been reported in literature, as such the experience with our propensity matched population with a 30-day mortality of 21.7% is comparable.^14,17–19^ The higher mortality rate can be attributed to the high incidence of concomitant procedures as well as the non-elective setting of more than 50% of the operations. Combined procedures have been previously identified as independent risk factor in other studies^19,20^ associated with an increased mortality of up to 56%.^15,16,21^ Secondly, it has also been shown that urgent and emergent conditions are an independent predictor of mortality^18,19^ and that conversely elective timing is a protective factor for early mortality.^20^

The previously mentioned factors are partly reflected by a higher EuroSCORE I of 14.6 ± 12.2 in comparison to recently published studies in which it ranged between 8.2 and 11.8.^16,18–20,22^ An additional risk factor for hospital mortality that finds no consideration in the EuroSCORE I, but was identified as such by Taamallah and colleagues,^17^ is time spent on dialysis exceeding 54 months. Here, again our population was considerably longer on dialysis (59 ± 52 months, 68 ± 54 and 50 ± 49 for MiECC and CECC respectively) than similarly published patient collectives with times on dialysis ranging between 36 and 48 months^16–18^

Clamping and bypass durations were noticeably shorter in the MiECC population, but this difference was not statistically significant, as such we postulate that this effect is not sufficient to explain a significant difference in outcome. A higher incidence of combined procedures in the CECC group (11 (37%) vs. 8 (27%) patients, *p* = .405), could explain this difference.

Ellam et al already demonstrated that MiECC reduces intraoperative transfusions of red blood cell concentrates.^1^ We could confirm this observation in our study with MiECC 1.4 ± 1.8 units vs CECC 2.8 ± 1.7, *p* < .001. Interestingly, this advantage of MiECC could not be sustained during the rest of the hospital stay. Although minimized systems reduce SIRS, it cannot completely negate the physiological trauma of extracorporeal circulation, and thus postoperative inflammation occurs along with increased vascular permeability and concomitant hemodilution.^23^ This is especially true for patients on dialysis, who often suffer from anemia and uremia due to the underlying disease and belong to a group of patients who are more likely to experience a liberal transfusion strategy. Interestingly most studies that have found significant differences in transfusion requirements rather adopted a restrictive transfusion strategy with a clear transfusion trigger.^1,2,24,25^

The benefits of MiECC over CECC have already been demonstrated in high-risk patients.^26^ Although there was no significant difference in the frequency of adverse events following propensity score matching between the two groups (MiECC 9 (30%) vs CECC 13 (43%), *p* = .284), it is noteworthy, that there has been no low cardiac output event in the MIECC group while the incidence was 17% in the CECC group (*p* = .052).

There is a significant difference in the 30-day postoperative mortality in favour of the MiECC group (3 (10%) vs 10 (33%), *p* = .028. For this result, one must acknowledge the difference in EuroSCORE I (MiECC 12.3 ± 10.4 vs CECC 16.9 ± 13.5, *p* = .132), although not significant and the number of combined cases, which slightly favour the MiECC group. However, it must be mentioned that 50% of the mortalities in the CECC occurred in the patients who had an isolated CABG procedure. Therefore, the greater role in this significant difference in mortality in our opinion lies in a better preservation of homeostasis due to a reduced physiological trauma, which is the key stone to a MiECC philosophy. This is reflected in reduced inflammation, reduced hemodilution, reduced coagulopathy as well as a better myocardial protection, which is reflected by a lower incidence of low cardiac output events.^11^

To our best of knowledge this study is the first comparative research between MiECC and CECC on dialysis-dependent patients undergoing cardiac surgery.

## Limitations

This study has several limitations. Foremost it is limited by its retrospective nature. However, to compensate for the lack of randomization, a propensity score matching was employed. Additionally, we can only provide early postoperative outcomes, a longer follow-up in this population regarding long-term survival and quality of life between MiECC and CECC would be of great interest.

## Conclusion

This study emphasizes the idea that the benefits of MiECC could have a greater impact in high-risk patient populations with depleted physiologic reserves, including patients on dialysis. They hence warrant further research into the use of MiECC in patients with end stage renal disease as well as in other high risk patient populations.

## Supplemental Material

Supplemental Material - Influence of minimal invasive extracorporeal circuits on dialysis dependent patients undergoing cardiac surgery Supplemental Material for Influence of minimal invasive extracorporeal circuits on dialysis dependent patients undergoing cardiac surgery by Thai Duy Nguyen, Mohammed Morjan, Khaldoun Ali, Ingo Breitenbach, Wolfgang Harringer and Aschraf El-Essawi in Perfusion
