# Supplementary material for: Influence of minimal invasive extracorporeal circuits on dialysis dependent patients undergoing cardiac surgery
Source: Perfusion. 2023 Nov 17;39(8):1708–14. doi: 10.1177/02676591231216794 (PMC11492210; doi:10.1177/02676591231216794)
Supplement: Supplemental Material - Influence of minimal invasive extracorporeal circuits on dialysis dependent patients undergoing cardiac surgery [file sj-pdf-1-prf-10.1177_02676591231216794.pdf]

Supplement

Table S1. Adverse events pre matching

Variable	All Patients = 131	MiECC = 63	CECC = 68	p-value
Myocardial infarction	1	0	1	0.334
Reoperation	10	2	8	0.064
Pneumonia	8	4	4	0.911
Stroke	4	4	0	0.051
Mediastinitis	1	0	1	0.334
Resuscitation	6	3	3	0.924
Sepsis	5	2	3	0.699
Low cardiac output	8	0	8	0.034
Postoperative delirium	14	8	6	0.473

Data are presented as number.

Table S2. Results pre matching

Variable	All Patients = 131	MiECC = 63	CECC = 68	p-value
Operating time	234 ± 102	209 ± 77	258 ± 116	0.005
Clamping time	78 ± 48	58 ± 32	97 ± 53	<0.001
Bypass time	128 ± 81	101 ± 51	153 ± 95	<0.001
Transfused RBC units (IO)	2.3 ± 2.2	1.2 ± 1.5	3.3 ± 2.4	<0.001
Transfused RBC units (total)	4.8 ± 4.8	3.7 ± 3.7	5.9 ± 5.5	0.001
Transfused FFP units (IO)	1.3 ± 2.4	0.6 ± 1.4	1.87 ± 2.9	<0.001
Transfused FFP units (total)	4.1 ± 6.7	3.6 ± 7.1	4.5 ± 6.3	0.058
Transfused platelet units (IO)	0.3 ± 0.7	0.2 ± 0.5	0.4 ± 0.8	0.162
Transfused platelet units (total)	0.5 ± 1.0	0.3 ± 0.8	0.6 ± 1.2	0.179
30-day mortality	27 (20.6)	8 (12.7)	19 (27.9)	0.031

Data are presented as mean ± SD or number (%). Times in minutes. IO = Intraoperative. Total = intraoperative + postoperative. RBC = Red blood cell. FFP = Fresh frozen plasma concentrate
